# Hydrodynamic Shape Changes Underpin Nuclear Rerouting in Branched Hyphae of an Oomycete Pathogen

**DOI:** 10.1128/mBio.01516-19

**Published:** 2019-10-01

**Authors:** Edouard Evangelisti, Liron Shenhav, Temur Yunusov, Marie Le Naour–Vernet, Philipp Rink, Sebastian Schornack

**Affiliations:** a Sainsbury Laboratory Cambridge University (SLCU), Cambridge, United Kingdom; University of British Columbia

**Keywords:** oomycetes, *Phytophthora palmivora*, nucleus movement, centrosome, hydrodynamics

## Abstract

Multinucleate fungi and oomycetes are phylogenetically distant but structurally similar. To address whether they share similar nuclear dynamics, we carried out time-lapse imaging of fluorescently labeled Phytophthora palmivora nuclei. Nuclei underwent coordinated bidirectional movements during plant infection. Within hyphal networks growing *in planta* or in axenic culture, nuclei either are dragged passively with the cytoplasm or actively become rerouted toward nucleus-depleted hyphal sections and often display a very stretched shape. Benomyl-induced depolymerization of microtubules reduced active movements and the occurrence of stretched nuclei. A centrosome protein localized at the leading end of stretched nuclei, suggesting that, as in fungi, astral microtubule-guided movements contribute to nuclear distribution within oomycete hyphae. The remarkable hydrodynamic shape adaptations of *Phytophthora* nuclei contrast with those in fungi and likely enable them to migrate over longer distances. Therefore, our work summarizes mechanisms which enable a near-equal nuclear distribution in an oomycete. We provide a basis for computational modeling of hydrodynamic nuclear deformation within branched tubular networks.

## Abstract (precis)

Despite their fungal morphology, oomycetes constitute a distinct group of protists related to brown algae and diatoms. Many oomycetes are pathogens and cause diseases of plants, insects, mammals, and humans. Extensive efforts have been made to understand the molecular basis of oomycete infection, but durable protection against these pathogens is yet to be achieved. We use a plant-pathogenic oomycete to decipher a key physiological aspect of oomycete growth and infection. We show that oomycete nuclei travel actively and over long distances within hyphae and during infection. Such movements require microtubules anchored on the centrosome. Nuclei hydrodynamically adapt their shape to travel in or against the flow. In contrast, fungi lack a centrosome and have much less flexible nuclei. Our findings provide a basis for modeling of flexible nuclear shapes in branched hyphal networks and may help in finding hard-to-evade targets to develop specific antioomycete strategies and achieve durable crop disease protection.

## INTRODUCTION

Although nuclei are usually depicted as immobile, their movement is essential for the growth and development of all eukaryotes, including those with filamentous morphology (1). The nuclear dynamics of fungi have been studied during development in axenic culture as well as during infection of susceptible hosts (2). Nuclear movements may also contribute to infection success (3–6). Recent work in Magnaporthe oryzae documented the migration of nuclei into the appressorium and, later, into the primary infectious hypha upon rice infection (4).

Fungal nuclei often distribute equally within hyphae, such as in Aspergillus nidulans germ tubes upon asexual spore germination (7). This involves microtubules, cytoplasmic dynein, and components of the dynactin complex. Indeed, treatment with the microtubule-depolymerizing drug Benomyl or mutations in beta-tubulin alter nuclear distribution in A. nidulans (8). Similar nuclear distribution defects are observed when Neurospora crassa cytoplasmic dynein genes (9) or the dynactin component *Arp1* (10) is mutated. Nuclear distribution is also affected in the Ashbya gossypii dynein null mutant, but nuclei accumulate at hyphal tips rather than at the spore end (11).

Studies in budding yeast (Saccharomyces cerevisiae) showed that proper orientation of the mitotic spindle during anaphase is crucial for the mother cell and the bud to receive a single nucleus after nuclear division (12). Such orientation is achieved through astral microtubules (12). Assembly of a functional mitotic spindle also requires precise coordination between the nuclear cycle and the major microtubule organizing center, also known as the centrosome (13). A canonical centrosome is composed of two centrioles surrounded by an electron-dense protein-containing matrix (pericentriolar matrix), which nucleates microtubules (14). All fungi except Chytridiomycota possess an acentriolar centrosome termed the spindle pole body (SPB) (14, 15). The SPB is embedded in the nuclear envelope and nucleates spindle microtubules inside the nucleus as well as astral microtubules toward the cytoplasmic side (14). The growing ends of microtubules are occupied by dynein. In *A. gossypii*, dynein capture by the cortical nuclear migration protein Num1 initiates the pulling of cytoplasmic microtubules, thereby moving the attached nucleus (16). Orthologs of *Num1* include the *AND1* gene from *M. oryzae* (5) and *ApsA*/*ApsB* from A. nidulans (17).

Oomycetes are a group of microorganisms with filamentous morphologies that are phylogenetically distant from fungi. They differ from fungi by structural, biochemical, and genetic features such as genome size, ploidy, cell wall composition, pigmentation, secondary metabolites, and the chemical nature of mating hormones and reserve compounds (18). Plant-pathogenic oomycetes cause devastating diseases worldwide that impact crop yield, threaten food security, and damage natural ecosystems (19, 20). For instance, blight, canker, and rot diseases caused by plant-infecting oomycetes from the genus *Phytophthora*, such as the Irish Famine pathogen Phytophthora infestans and its tropical relative Phytophthora palmivora, cause multimillion-dollar losses yearly (21–23). Whether oomycetes display similar nuclear dynamics as filamentous fungi remains to be addressed. Pioneering work based on staining methods revealed elongated nuclei, suggesting a potential movement in cysts of the diatom-infecting oomycete Lagenisma coscinodisci (24) as well as in hyphae and germinating cysts of *P. infestans* (25, 26). Similarly to fungi, oomycete nuclei are known to distribute equally within coenocytic (aseptate) hyphae, and this distribution is altered by the actin polymerization inhibitor latrunculin B (27–29) or by silencing the Phytophthora capsici cell cycle regulator homologue *sda1* (30).

We used time-lapse imaging to investigate nuclear dynamics of *P. palmivora* during cyst germination and subsequent root and leaf infection in addition to axenically cultured hyphae. For this purpose, we generated a versatile toolbox for efficient dual labeling of oomycete hyphae and organelles that enabled the rapid testing of various promoter-reporter constructs. We found that *P. palmivora* nuclei undergo coordinated bidirectional movements during plant infection and actively or passively move during mycelial growth to achieve a near-equal distribution of nuclei within hyphae. Active movement of individual nuclei frequently resulted in dramatic alterations of near-globular nuclei into extensively stretched shapes. Shape changes often cooccurred with a rerouting of nuclei toward nucleus-depleted hyphal sections. The centrosome-labeling Centrin2 protein occupied the trailing end of near-globular nuclei and the leading end of stretched nuclei. Furthermore, the microtubule polymerization inhibitor Benomyl decreased nuclear stretching and rerouting. Hence, our results unravel commonalities and differences in the dynamics of oomycete and fungal nuclei. Together, this constitutes the basis for new models of nuclear migration and hydrodynamic deformability that may ultimately offer alternative strategies to tackle oomycete diseases.

## RESULTS

### Bidirectional nuclear movements occur during *P. palmivora* cyst germination.

To investigate nuclear dynamics during *P. palmivora* cyst germination, we generated the pTORKRm43GW Gateway vector, which allows for dual labeling of hyphae and organelles (see Fig. S1 and S2 and Table S1A in the supplemental material), and transformed the *P. palmivora* isolate LILI (P16830). The vector contains a cassette for the Ham34 promoter-driven constitutive expression of a cytoplasmic tdTomato fluorescent reporter in addition to a cyan nucleus-localized monomeric TFP1 (NLS:mTFP1) expressed under the *P. palmivora ubiquitin-conjugating enzyme 2* (*UBC2*) native promoter (Fig. S3 and S4). Among seven independent transformants, two showed distinct hyphal populations expressing either tdTomato or mTFP1 reporters, while five showed both cytoplasmic tdTomato (td) fluorescence and nuclear mTFP1 (NT) fluorescence. The latter were used to obtain the transgenic *P. palmivora* line LILI-td-NT used in this study (Fig. S3).

10.1128/mBio.01516-19.1 FIG S1 Schematic representation of the pTOR-Gateway. A multisite attR4/attR3 Gateway insertion cassette allows for rapid testing of multiple promoter-reporter constructs. Adjacent is a cassette for constitutive expression of a cytoplasmic fluorescent reporter (either mTFP1, mWasabi, mCitrine, or tdTomato) under the control of *Bremia lactucae* promoter Ham34. Vectors carry neomycin phosphotransferase (nptII) selectable marker. Download FIG S1, PDF file, 0.2 MB. Copyright © 2019 Evangelisti et al. 2019 Evangelisti et al. This content is distributed under the terms of the Creative Commons Attribution 4.0 International license.

10.1128/mBio.01516-19.2 FIG S2 Growth habit of transgenic *P. palmivora* strains carrying empty pTOR-Gateway vectors on N. benthamiana leaves. (A to D) Leaves from 4-week-old N. benthamiana plants were inoculated with mycelium plugs of transgenic *P. palmivora* strains carrying empty pTOR-Gateway vectors. Fluorescence was monitored within leaf tissues after 2 days. Representative images of areas infected with mycelium expressing mTFP1 (A), mWasabi (B), mCitrine (C), and tdTomato (D) are shown. Yellow asterisks indicate haustoria. Bar, 10 μm. Download FIG S2, PDF file, 1.5 MB. Copyright © 2019 Evangelisti et al. 2019 Evangelisti et al. This content is distributed under the terms of the Creative Commons Attribution 4.0 International license.

10.1128/mBio.01516-19.3 FIG S3 Dual labeling of *P. palmivora* hyphae and nuclei. (A) Schematic view of the construct used for dual labeling of nuclei and hyphae in *P. palmivora* strain LILI-td-NT. Backbone elements are not represented. (B) Representative pictures of a transformant expressing tdTomato as well as a nucleus-localized mTFP1 (NLS:mTFP1) driven by the *P. palmivora ubiquitin-conjugating enzyme 2* (*UBC2*) native promoter. Representative pictures of transformants expressing both markers as well as rare cases of transformants expressing either tdTomato or mTFP1 in distinct hyphae. Bar, 10 μm. Download FIG S3, PDF file, 1.0 MB. Copyright © 2019 Evangelisti et al. 2019 Evangelisti et al. This content is distributed under the terms of the Creative Commons Attribution 4.0 International license.

10.1128/mBio.01516-19.4 FIG S4 *UBC2* transcript levels during N. benthamiana root infection. N. benthamiana roots were inoculated with zoospores from the transgenic *P. palmivora* strain ARI-tdTomato and harvested at different times corresponding to early infection (3 to 6 h), biotrophy (18 to 24 hai), and necrotrophy (30 to 48 hai). Expression data are given relative to *P. palmivora WS21* and *EF1α* reference genes. Statistical significance was assessed using one-way analysis of variance and Tukey’s honestly significant difference test (*P < *0.05). MZ, axenically grown mycelium with sporangia. Download FIG S4, PDF file, 0.2 MB. Copyright © 2019 Evangelisti et al. 2019 Evangelisti et al. This content is distributed under the terms of the Creative Commons Attribution 4.0 International license.

10.1128/mBio.01516-19.10 TABLE S1 (A) pTOR-Gateway vectors. pTOR-Gateway vectors follow naming conventions used for Gateway vectors. K indicates *nptII*, while C, F, Y, and R stand for cyan, green, yellow, and red fluorescence, respectively. The multisite Gateway cassette carries attR4 and attR3 sites and hence was arbitrarily named m43GW in the absence of a T-DNA left border to define cassette orientation. (B) Primers used in this study. Download Table S1, PDF file, 0.2 MB. Copyright © 2019 Evangelisti et al. 2019 Evangelisti et al. This content is distributed under the terms of the Creative Commons Attribution 4.0 International license.

**FIG 1:**
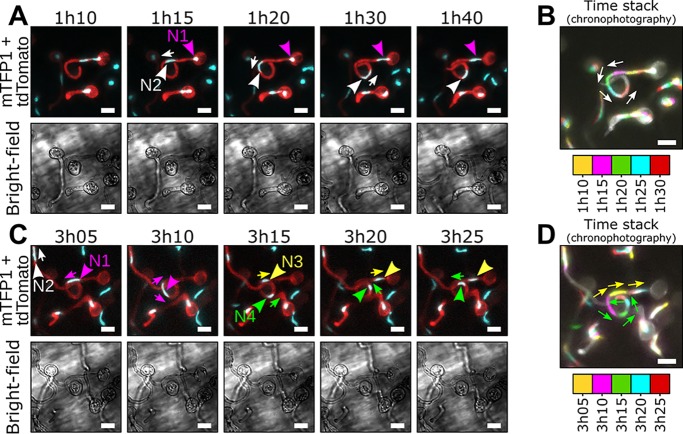
Repositioning of daughter nuclei follows division during P. palmivora cyst germination (video 1). (A to D) Time-lapse imaging of germinating cysts from a transgenic P. palmivora strain expressing cytoplasmic tdTomato as well as nucleus-localized mTFP1 (LILI-td-NT) during growth at the surface of N. benthamiana roots. (A) Following first nuclear division, daughter nucleus N1 (magenta arrowhead) migrates toward the tip of the germ tube (white arrow), while the second nucleus, N2 (white arrowhead), remains within the cyst body. (B) Chronophotographic view (time stack) of the previous sequence, showing the successive locations of nucleus N2. Time frames are color coded and overlaid. (C) Nuclear movements following N1 division. Daughter nuclei N3 (yellow arrowhead) and N4 (green arrowhead) move in opposite directions (yellow and green arrows, respectively) and distribute equally within the germ tube. (D) Chronophotographic view (time stack) of the sequence shown in panel C, showing opposite movement of nuclei N3 and N4. Bar, 10 μm.

We then incubated encysted LILI-td-NT zoospores in a liquid compartment containing a Nicotiana benthamiana seedling (Fig. S5) and monitored nuclear movements during the growth of cyst germ tubes (Fig. 1A and B, video 1 [https://zenodo.org/record/3378706/files/Video1.mp4]). Germ tubes emerged from mononucleate cysts and were initially devoid of a nucleus. After 1 h when germ tubes were longer than 10 μm on average, the sole nucleus became stretched and moved from the cyst into the germ tube while the cyst remained filled with cytoplasm (Fig. 1A, video 1). The nucleus divided in the germ tube, but no leakage of mTFP1 fluorescence from the nucleus was detectable during division. The daughter nucleus N1 subsequently retracted to the cyst body, while the second daughter nucleus, N2, moved forward within the germ tube at an average speed of 0.02 μm/s (Fig. 1A, video 1). Upon further extension of the germ tube, the cyst-resident nucleus N1 reentered the germ tube, still leaving cytoplasm in the cyst behind (Fig. 1A, video 1) and divided 3 h after encystment into daughter nuclei N3 and N4 (Fig. 1B, video 1). N3 and N4 moved away from each other, with nucleus N3 moving toward the cyst body and nucleus N4 moving forward within the germ tube. Collectively, these findings demonstrate that nuclei of germinating *P. palmivora* cysts divide in a closed mitosis and are then distributed evenly along the germ tube through concomitant, bidirectional movements.

10.1128/mBio.01516-19.5 FIG S5 Experimental setup used for time-lapse imaging of infected N. benthamiana roots. (A) Schematic representation of the experimental setup. A 1-week-old N. benthamiana seedling (2) is mounted in a liquid compartment (3) between a slide (1) and a coverslip (4). The edges of the coverslip are sealed with a 4:1 mix of paraffin and lanolin (5). The upper part of the coverslip is filled with water for use with a water-dipping objective. (B) Representative image of the experimental setup. Numbers refer to the same elements as described above. Download FIG S5, PDF file, 0.6 MB. Copyright © 2019 Evangelisti et al. 2019 Evangelisti et al. This content is distributed under the terms of the Creative Commons Attribution 4.0 International license.

### Appressorium differentiation alters nuclear dynamics in the germ tube.

**FIG 2:**
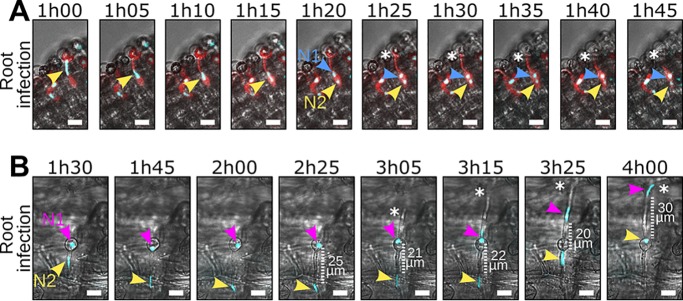
Appressorium differentiation alters *P. palmivora* nuclear dynamics in the germ tube. (A and B) Time-lapse imaging of N. benthamiana root infection by a *P. palmivora* LILI-td-NT strain (video 2). (A) Representative sequence of a successful infection event (video 2A). Arrowheads indicate nuclei. Asterisks indicate migration of the cytoplasm out of the cyst. (B) Representative sequence of an unsuccessful infection event, leading to the differentiation of a second germ tube (indicated by asterisks) opposite the first one (video 2B). Arrowheads indicate nuclei. Bar, 10 μm.

We then investigated nuclear movements during the onset of N. benthamiana root infection, using a similar setup as previously described (Fig. 2 and Fig. S5, video 2 [https://zenodo.org/record/3378706/files/Video2.mp4]). Consistent with previous observations, the cyst-resident nucleus moved into the germ tube 1 h after encystment and reached the swollen tip of a differentiating appressorium. However, unlike cyst germination, we did not observe backward movement of a daughter nucleus toward the cyst after nuclear division. Instead, the cyst was progressively depleted from cytoplasm during appressorium differentiation (Fig. 2A, video 2A).

In rare instances, appressorium differentiation did not occur (Fig. 2B, video 2B). Nuclear divisions and movements were then similar to those during cyst germination, where nucleus N2 moved toward the germ tube tip and N1 resided in the cyst. Upon infection failure, a second germ tube germinated from the cyst opposite the first after 3 h. Nucleus N1 then proceeded from the cyst into the new germ tube, while nucleus N2 simultaneously retreated into the cyst. A distance of 20 to 30 *μ*m was maintained between the two nuclei through the entire process (Fig. 2B, video 2B). Taken together, these observations suggest that successful appressorium differentiation influences nuclear distribution dynamics and that nuclei may be recalled and rerouted into additional infection attempts.

### Differential nuclear migration rates occur throughout the *P. palmivora* hyphal network both *in planta* and in axenic culture.

**FIG 3:**
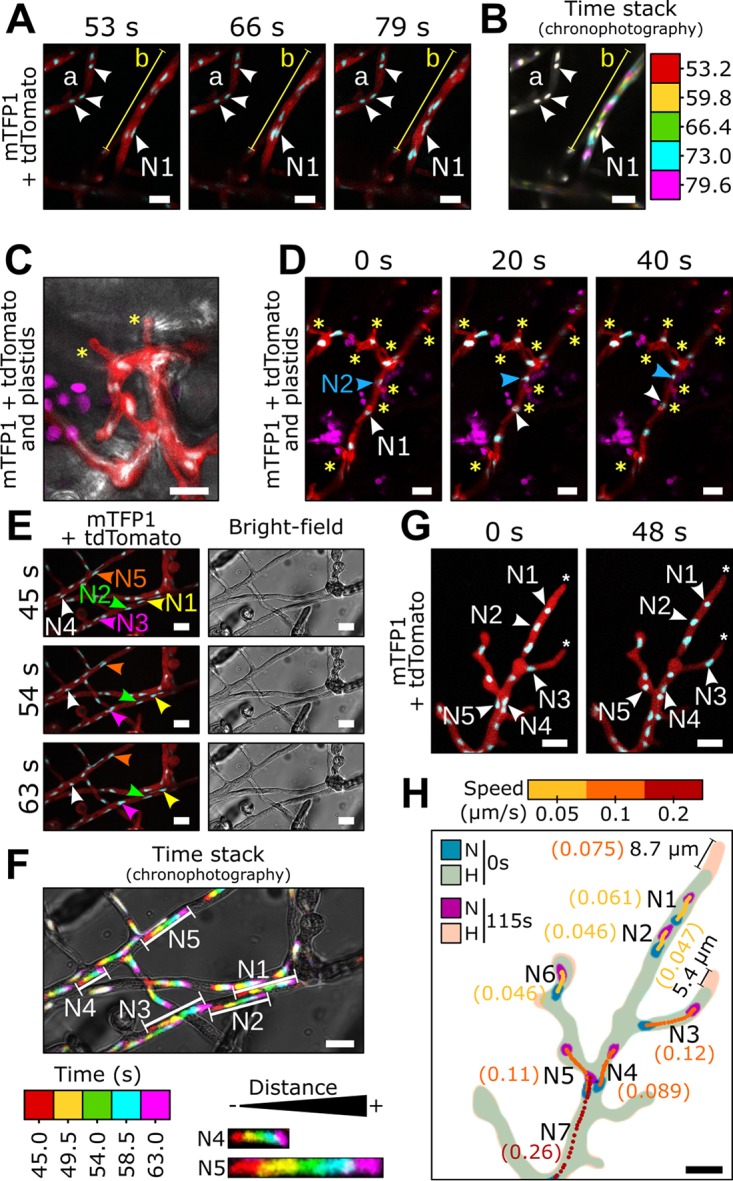
Differential nuclear migration rates occur throughout *P. palmivora* hyphal network. Time-lapse imaging of *P. palmivora* LILI-td-NT hyphae infecting an N. benthamiana leaf (A to D, videos 3 and 4) or growing axenically on V8 medium (E to H, videos 5 and 6). (A, video 3) Representative sequence of nuclear movements within infectious *P. palmivora* hyphae. (B, video 3) Chronophotographic display (time stack) of the sequence shown in panel A. The successive time frames are color coded and overlaid. (C) Representative sequence of *P. palmivora* haustoria (asterisks) on an N. benthamiana leaf. Arrowheads indicate nuclei. (D, video 4) Representative sequence of nuclear movements within haustoriated *P. palmivora* hyphae. (E, video 5) Representative sequence of nuclear migration on axenically grown mycelium. (F, video 5) Chronophotographic display of the sequence shown in panel E. (G, video 6) Representative sequence of nuclear movements near hyphal tips. (H) Nuclear trajectories for seven nuclei at the hyphal tip. Average speed is indicated in parentheses. Lines indicate hyphal segments. Arrowheads indicate individual nuclei. N, nuclei; H, hyphae. Bar, 10 μm.

During the biotrophic infection stage in living N. benthamiana leaves, *P. palmivora* nuclei moved rapidly within intracellular hyphae (Fig. 3A and B, video 3 [https://zenodo.org/record/3378706/files/Video3.mp4]) but did not enter haustoria within plant cells (Fig. 3C and D, video 4 [https://zenodo.org/record/3378706/files/Video4.mp4]). Most nuclei adopted a round shape, likely due to the diameter of hyphae (up to 6 μm, *n* = 10) being greater than the diameter of nuclei (3.5 μm, *n* = 10). In contrast, in germ tubes (2 μm, *n* = 10) nuclei remained elongated even when not moving (Fig. 1). Interestingly, independent hyphae showed differential nuclear migration rates, as evidenced by chronophotographic display of the time-lapse image series (Fig. 3B). For instance, a continuous flow of nuclei was observed in the hyphal segment b, while no movement could be detected in the hyphal segment a (Fig. 3A). Most nuclei were dragged passively by the bulk nuclear flow (P-type nuclei). Conversely, 10% of the nuclei, such as N1, stretched and maintained a near-constant location over time within segment b despite a continuous flow of nuclei bypassing them (Fig. 3B), which suggests that they are actively anchored (A-type nuclei). These findings demonstrate that different motion speeds occur throughout the hyphal network during biotrophy and that some nuclei may behave independently of the surrounding mass nuclear flow.

Nuclear movements during saprotrophic growth on a V8 agar plate (Fig. 3E to H, video 5 [https://zenodo.org/record/3378706/files/Video5.mp4]) were similar to those during plant infection, and again, differential nuclear motion speeds were observed within hyphal segments (Fig. 3E and F). For instance, nuclei N1 to N3 all moved with similar speed within the same hypha. In contrast, nucleus N5 moved faster than nuclei that moved passively with mass nuclear flow like N4. In addition, axenic hyphae allowed us to study hyphal tips (Fig. 3G and H, video 6 [https://zenodo.org/record/3378706/files/Video6.mp4]), where mass nucleus movement slowed and nucleus N7 (average speed, 0.26 μm/s) moved faster than downstream nuclei N1 to N6 (average speed ranging from 0.046 to 0.12 μm/s). Subapical zones were devoid of nuclei up to 9 μm (*n* = 10) from the hyphal tips (Fig. 3H). Again, some nuclei migrate at speeds different from mass nuclear movement, likely due to active anchoring (A-type nuclei).

### A-type nuclei stretch and move independently of surrounding nuclear flow.

**FIG 4:**
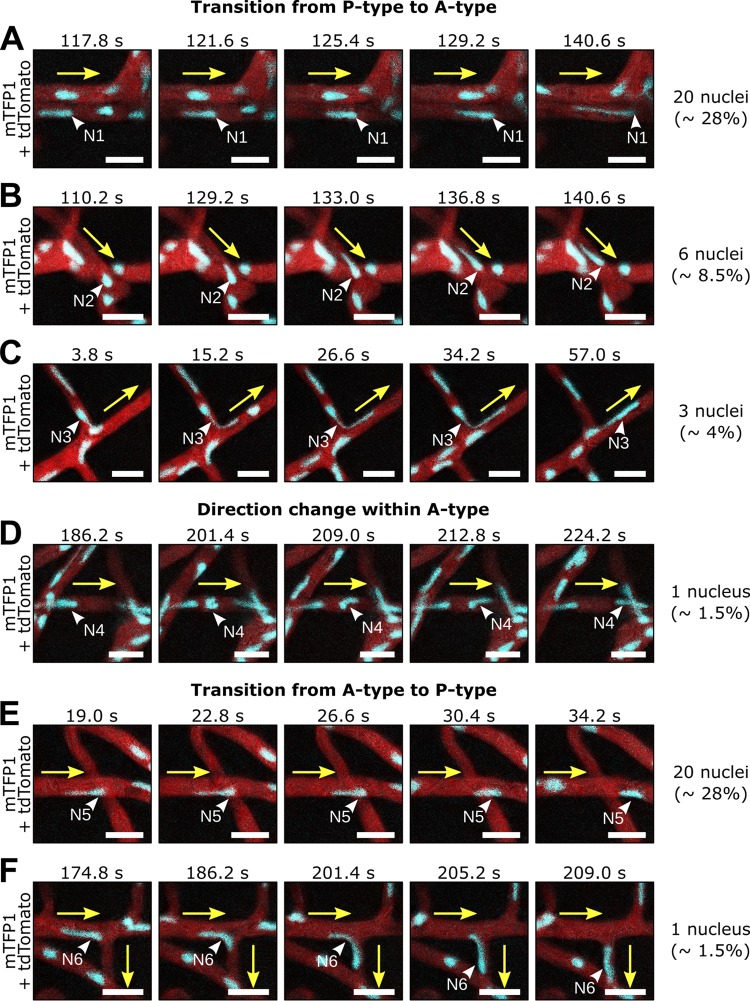
Individual *P. palmivora* nuclei stretch and move independently of surrounding nuclear flow. (A to F, video 6) Time-lapse imaging of axenically grown *P. palmivora* LILI-td-NT hyphae containing nuclei transitioning from passive (P-type) to active (A-type) movements (A to D) or, conversely, from A type to P type (E and F). (A) Nucleus slows down and stretches sternward. (B) Nucleus shrinks and starts moving together with the surrounding nuclear flow. (C) Same as panel B, but nucleus enters a branch. (D) Nucleus stretches and moves opposite the surrounding nuclear flow. (E) Same as panel D but entering a branch. (F) Nucleus stretches and moves opposite the surrounding nuclear flow and then tumbles and initiates a similar movement in the opposite direction. Yellow arrows indicate mass nuclear flow. White arrowheads indicate nuclei. Frequencies are given based on a total of 70 nuclei. Bar, 10 μm.

To gain more insight on the dynamics of A-type *P. palmivora* nuclei, we tracked nuclear stretching and other trajectories that differed from the mass nuclear movement and assessed their frequency within the hyphal network (Fig. 4, video 5). We identified several different behaviors of A-type nuclei. Frequently (28%), P-type nuclei transition into the A-type state and stop within the flow to either stay at a given position and stretch out sternward (Fig. 4A), suggesting that they are anchored at one point, or move against the flow (Fig. 4B, 8.5%) and into branches (Fig. 4C, 4%). In some rare cases (Fig. 4D, 1.5%), A-type nuclei moving against the flow changed their direction of movement by tumbling, such that the stretched part always pointed to the direction of the migration. With a frequency similar to the P- to A-type transition (28%), A-type nuclei reverted to passive movement (P type) where they often shrank to adopt a round shape (Fig. 4E). In rare cases (1.5%), such passive flow then also dragged these nuclei into side branches (Fig. 4F). In summary, nuclei can undergo transitions between passive and active movements that allow them to position themselves within the hyphal network. Active movement correlates with changes in nuclear shape, where nuclei get remarkably stretched.

Nuclear shape flexibility is often associated with the absence of lamin-encoding genes (2), but *laminA* has been identified in several *Phytophthora* species, including *P. palmivora* (31, 32). To test whether *P. palmivora* encodes a functional lamin A protein that controls nuclear shape, we generated transgenic strains expressing an *mCitrine*:*laminA* translational fusion under the control of either the constitutive Ham34 promoter or the native *laminA* promoter (Fig. S6). We found that expression of either construct resulted in a nucleoplasmic labeling with mCitrine. Notably, *mCitrine*:*laminA* overexpression triggered nuclear blebbing (Fig. S6A) and arrested hyphal growth at an early stage, while expression under the native promoter did not alter nuclear shape (Fig. S6B) and hyphal growth was indiscernible from the wild type. Therefore, *P. palmivora* lamin A may contribute to nuclear shape and integrity.

10.1128/mBio.01516-19.6 FIG S6 Generation of a *P. palmivora* lamin A reporter. Transformation of *P. palmivora* LILI with a construct for constitutive (A and B) or native (C and D) expression of an mCitrine:LamA-Cter reporter. (A) Schematic view of the construct used for constitutive or native expression of the lamin reporter together with a nucleus-localized mTFP1. Backbone elements are not represented. (B) Representative pictures of the bubbling phenotype observed upon constitutive expression of the lamin reporter. Arrowheads indicate bubbling nuclei. Bar, 10 μm. (C) Representative pictures of a hyphal segment upon native expression of the lamin reporter, showing absence of nuclear bubbling. Arrowheads indicate bubbling nuclei. Bar, 10 μm. Download FIG S6, PDF file, 2.3 MB. Copyright © 2019 Evangelisti et al. 2019 Evangelisti et al. This content is distributed under the terms of the Creative Commons Attribution 4.0 International license.

### Nuclear shape is altered upon acceleration and deceleration.

**FIG 5:**
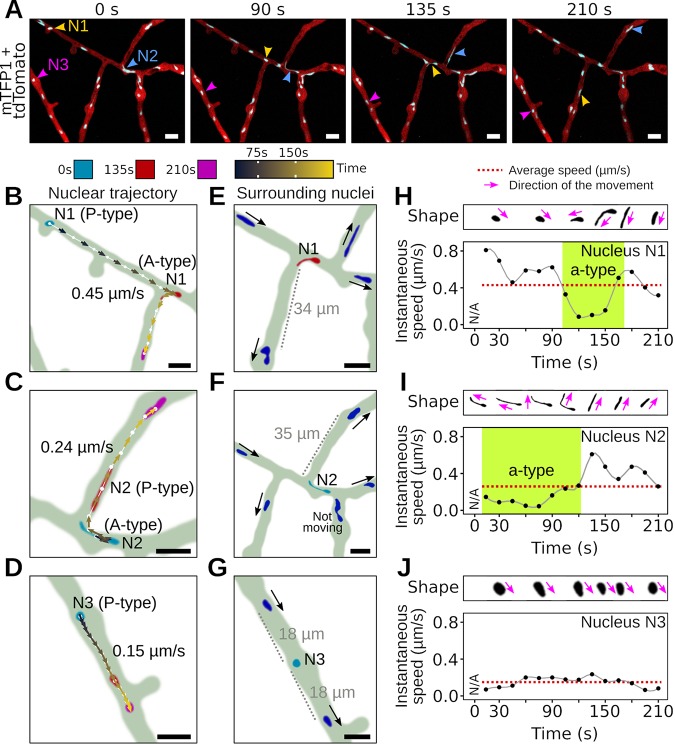
*P. palmivora* nuclear stretching correlates with rerouting toward nucleus-depleted hyphal segments. (A, video 7) Representative sequence of nuclear movements at a hyphal branch point, highlighting both A-type (nuclei N1 and N2) and P-type (nucleus N3) movements. Arrowheads indicate nuclei. (B to J) Analysis of nuclear speed and trajectories for nuclei N1 to N3. (B, E, and H) Maps of nuclear content in the vicinity of nuclei N1 (B), N2 (E), and N3 (H). Black arrows indicate nuclear movement. (C, F, and I) Nuclear trajectories of N1 (C), N2 (F), and N3 (I). Locations of nuclei N1 to N3 at first (0 s), intermediate (135 s), and last (210 s) time points are shown in blue, red, and magenta, respectively. Nuclear centroids are represented as dots. Arrows indicate instantaneous speed and direction of the movement. Average nucleus speed is indicated on the graph. (D, G, and J) Variation of instantaneous speed with time for nuclei N1 (D), N2 (G), and N3 (J). Average speed in shown as a red dotted line. Representative pictures of nucleus shape over time are shown above the graphs with arrows indicating direction of the movement. Bar, 10 μm.

To better understand how *P. palmivora* nuclei transition into A-type trajectories, we monitored the speed, shape, and location of nuclei neighboring individual nuclei at a hyphal branch point where the transition of P to A type may occur (Fig. 5, video 7 [https://zenodo.org/record/3378706/files/Video7.mp4]). We found that transitions between active and passive movement were frequently associated with change in nuclear shape (Fig. 5B to D) and facilitated a more equal nuclear distribution to fill empty areas (Fig. 5E to G). For instance, nucleus N1 initially followed a P-type behavior and then stretched and adopted an A-type behavior, eventually moving backward into the branch point (Fig. 5B) toward a 34-μm-long hyphal segment devoid of nuclei (Fig. 5E). Similarly, the stretched, A-type nucleus N2 moved backward into another branch point (Fig. 5C), reaching a 35-μm-long nucleus-depleted hyphal segment (Fig. 5F). In contrast, the P-type nucleus N3 maintained a round shape while moving (Fig. 5D), and an equal distance of 18 μm was maintained from the surrounding nuclei (Fig. 5G). Whether nucleus N3 is part of the same hypha as nuclei N1 and N2 could not be determined from our data set (Fig. 5A, video 7).

We then measured instantaneous speed and direction of nuclei undergoing significant shape changes (Fig. 5H to J). We found that the onset of shape change in A-type nuclei correlated with changes in their instantaneous speed and that shape was affected by the direction of movement. For instance, nucleus N1 stretched while undergoing a 10-fold speed decrease from 0.8 μm/s down to 0.08 μm/s and reverted to a round shape after speed increased back to 0.4 μm/s (Fig. 5H). Similarly, stretched nucleus N2 had an initial speed of 0.1 μm/s and later became more round after speed increased (Fig. 5I). In contrast, the P-type nucleus N3 that maintained a steady speed did not change its shape (Fig. 5J). Taken together, these results suggest that trajectories of A-type nuclei lead to their reallocation of nuclei into nucleus-depleted hypha sections. The cooccurrence of nuclear stretching during these reallocations suggests that forces act most prominently at a single nuclear envelope pole.

### A-type nuclear movement is impaired by the microtubule polymerization inhibitor Benomyl.

**FIG 6:**
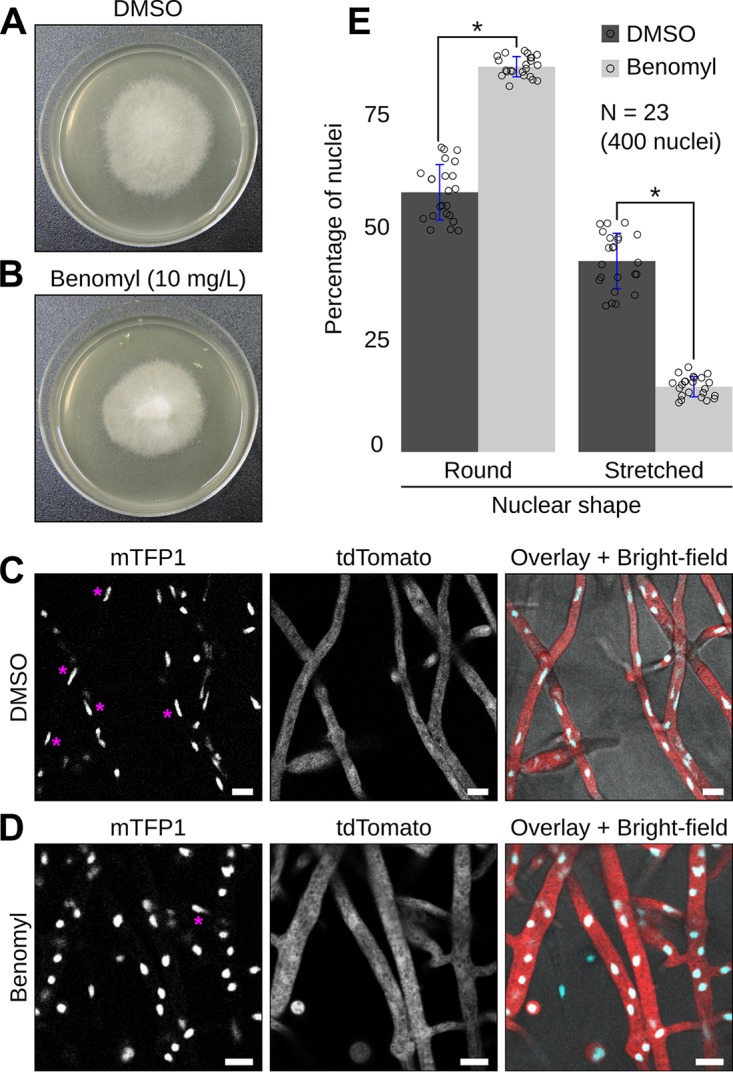
Antimicrotubule drug Benomyl impairs *P. palmivora* A-type nuclear movements. (A and B) Growth habit of transgenic *P. palmivora* LILI-td-NT mycelium growing on V8 agar plates without (dimethyl sulfoxide [DMSO] control) (A) or with (B) 10 mg/liter Benomyl. (C and D) Representative pictures of *P. palmivora* LILI-td-NT hyphae and nuclei in absence (C, video 8A) or presence (D, video 8B) of 10 mg/liter Benomyl. Bar, 10 μm. (E) Quantification of round (P-type) and stretched (A-type) nuclei within *P. palmivora* LILI-td-NT hyphae (*n* = 23, 400 nuclei). Statistical significance was assessed using the Wilcoxon test for paired samples (*P < *0.05).

Since microtubules are required for nuclear movements in fungi (2, 33), we assessed the effect of the microtubule polymerization inhibitor Benomyl on *P. palmivora* nuclei (Fig. 6, video 8 [https://zenodo.org/record/3378706/files/Video8.mp4]). Benomyl restricted *P. palmivora* growth in a dose-dependent manner. While 50 mg/liter Benomyl arrested hyphal growth on a V8 agar plate and resulted in abnormally shaped hyphae (Fig. S7), 10 mg/liter Benomyl barely reduced *P. palmivora* growth (Fig. 6A and B) and did not alter hyphal shape (Fig. 6C and D). Thus, 10 mg/liter Benomyl can be used to assess *P. palmivora* nuclear movements independently of changes in overall growth rate and hyphal anatomy. A near-equal distribution of nuclei was maintained along hyphae, suggesting that such a concentration did not have a dramatic effect on *P. palmivora* physiology. Time-lapse imaging showed nuclear movements in Benomyl-treated hyphae (Fig. 6C to E, video 8B). However, only 14% of nuclei displayed A-type behavior upon Benomyl treatment, compared to 42% in the control (*n* = 23, 400 nuclei) (Fig. 6E, video 8A). Instead, most nuclei (86%) were round and followed P-type trajectories, compared to 58% in the control (Fig. 6E). Taken together, these findings suggest that microtubules are required for A-type nuclear movements in *P. palmivora*.

10.1128/mBio.01516-19.7 FIG S7 Effect of antimicrotubule drug Benomyl on *P. palmivora* growth. (A) Representative pictures of *P. palmivora* LILI-td-NT mycelium growing on V8 agar plates supplemented or not with 10, 50, or 100 mg/liter Benomyl. (B) Confocal imaging of *P. palmivora* LILI-td-NT hyphae grown on V8 agar plates supplemented with 50 mg/liter Benomyl. Bar, 10 μm. Download FIG S7, PDF file, 0.9 MB. Copyright © 2019 Evangelisti et al. 2019 Evangelisti et al. This content is distributed under the terms of the Creative Commons Attribution 4.0 International license.

### The centrosome-associated protein Centrin2 localizes at the stretched extremities of nuclei.

**FIG 7:**
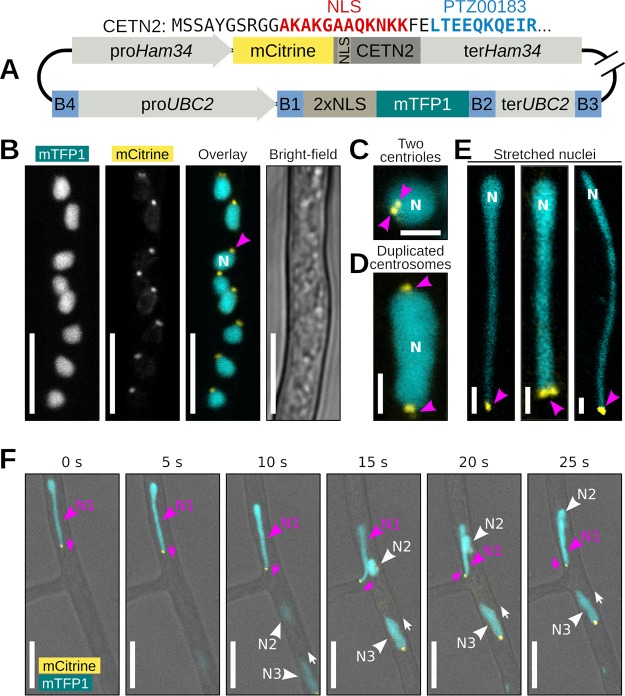
Centrin2 localizes to *P. palmivora* nuclear stretches. (A) Schematic view of the construct used for dual labeling of nuclei and centrosomes in *P. palmivora* strain LILI-NT-Ce. Backbone elements are not represented. (B, video 9) Representative picture of mCitrine-labeled Centrin2 (CETN2) within *P. palmivora* LILI-NT-Ce hyphae. (C) CETN2 localizes in two adjacent dots at the periphery of the nucleus. (D) Centrosome duplication in dividing nuclei. (E) Representative pictures of stretched *P. palmivora* nuclei (up to 30 μm long), with CETN2 localizing at the tip of the stretched areas. (F) Time-lapse imaging of nuclear movements at hyphal branch point. Nucleus N1 (magenta) follows an A-type trajectory backward to the branch point, while nuclei N2 and N3 follow a P-type trajectory. CETN2 localizes at the tip of the stretched part of nucleus N1, while CETN2 localizes at the back of nuclei N2 and N3. Bar, 2 μm (C to E) or 10 μm (B and F).

Fungal research has demonstrated that cell cortex-anchored dynein pulls the astral microtubules nucleated from the SPBs to move nuclei (2, 33). Centrosomes and centrioles which could serve as SPB equivalents in oomycetes have been reported for several *Phytophthora* species (34). We therefore labeled *P. palmivora* centrosomes using a fluorescently tagged centriolar lumen protein, Centrin2 (CETN2). To that end, we generated a transgenic *P. palmivora* LILI-NT-Ce strain by replacing the cytoplasmic tdTomato cassette from the previous dual reporter construct with an mCitrine fluorescent reporter fused N terminally to *P. palmivora CETN2* coding sequence (Ce) and monitored fluorescence over time at branching hyphae (Fig. 7, video 9 [https://zenodo.org/record/3378706/files/Video9.mp4]). CETN2 is composed of a nuclear localization signal followed by a Ca^2+^-binding protein/EF-hand superfamily protein domain (PTZ00183) (Fig. 7A). Expression of the CETN2/nucleus dual reporter led to labeling of nuclei with two adjacent punctate structures at the nuclear periphery (Fig. 7B), likely corresponding to the two centrioles of the *P. palmivora* centrosome (Fig. 7C). Similar labeling was obtained with the constitutive Ham34 promoter as well as the native *P. palmivora CETN2* promoter (Fig. S8). In addition, up to 15% of observed nuclei within a given region showed two sets of punctate structures located opposite each other, presumably as a result of centrosome duplication prior to nuclear division (Fig. 7D and Fig. S9).

10.1128/mBio.01516-19.8 FIG S8 Generation of a *P. palmivora* Centrin 2 (CETN2) reporter. (A) Schematic view of the construct for Ham34-promoter-driven expression of an mCitrine:CETN2 reporter together with a nucleus-localized mTFP1. Backbone elements are not represented. (B) Confocal imaging of a sporangium from a *P. palmivora* LILI-NT-Ce transgenic strain expressing the construct shown in panel A. Bar, 10 μm. (C) Schematic view of the construct for CETN2-promoter-driven expression of an mScarlet:CETN2 reporter together with a constitutively expressed nucleus-localized mTFP1. (D) Confocal imaging of a sporangium from a transgenic *P. palmivora* strain expressing the construct shown in panel C. Bar, 10 μm. Download FIG S8, PDF file, 0.8 MB. Copyright © 2019 Evangelisti et al. 2019 Evangelisti et al. This content is distributed under the terms of the Creative Commons Attribution 4.0 International license.

10.1128/mBio.01516-19.9 FIG S9 Frequency of centrosome duplication within *P. palmivora* hyphae. (A and B) Representative pictures of axenically grown hyphae from the transgenic *P. palmivora* LILI-NT-Ce strain growing on V8 medium. (A) Distribution of nuclei and Centrin2 (CETN2)-labeled centrosomes within a hyphal segment. Asterisks indicate nuclei with duplicated centrosomes. (B) Magnified views of nuclei 13 and 14. Bar, 10 μm. Download FIG S9, PDF file, 0.8 MB. Copyright © 2019 Evangelisti et al. 2019 Evangelisti et al. This content is distributed under the terms of the Creative Commons Attribution 4.0 International license.

We then specifically looked for elongated nuclei within *P. palmivora* hyphae and found several instances of *P. palmivora* nuclei stretching up to 30 μm in length. In all cases, mCitrine:CETN2-labeled punctate structures occurred at the very tip of the nuclear stretch (Fig. 7E). We then investigated the relationships between nuclear movements and positioning of mCitrine:CETN2-labeled punctate structures. We found that migrating P-type nuclei had their punctate structures oriented sternward, that is, opposite the direction of movement (nuclei N2 and N3) (Fig. 7F, video 9). In contrast, stretched nuclei moving against the mass nuclear flow had their punctate structures oriented in front (nucleus N1) (Fig. 7F). Our results suggest that *P. palmivora* nuclei are highly deformable and that mCitrine:CETN2-labeled punctate structures may be involved in microtubule-aided nuclear anchorage within hyphae and their movement against cytoplasmic flow.

## DISCUSSION

We studied the broad-host-range plant pathogen *Phytophthora palmivora* as a system to elucidate oomycete nuclear dynamics. By combining live imaging and a dual reporter expression system, we provide evidence for both active and passive nuclear movements within multinucleate, coenocytic hyphae. Different nuclear migration rates occur within the same hyphal segments, and nuclei move at a reduced rate in subapical regions compared to other hyphal segments, suggesting that the overall nuclear movement is influenced by cytoplasmic flow toward the mycelium periphery. Consistent with this hypothesis, nuclear migration in the fungus Neurospora crassa involves cytoplasmic flow (35, 36) and can be reversed by application of osmotic gradients across the colony (37). However, in contrast to observations made on Aspergillus nidulans (38), we did not observe a saltatory movement of nuclei, i.e., periodic inversion of the mass nuclear flow.

**FIG 8:**
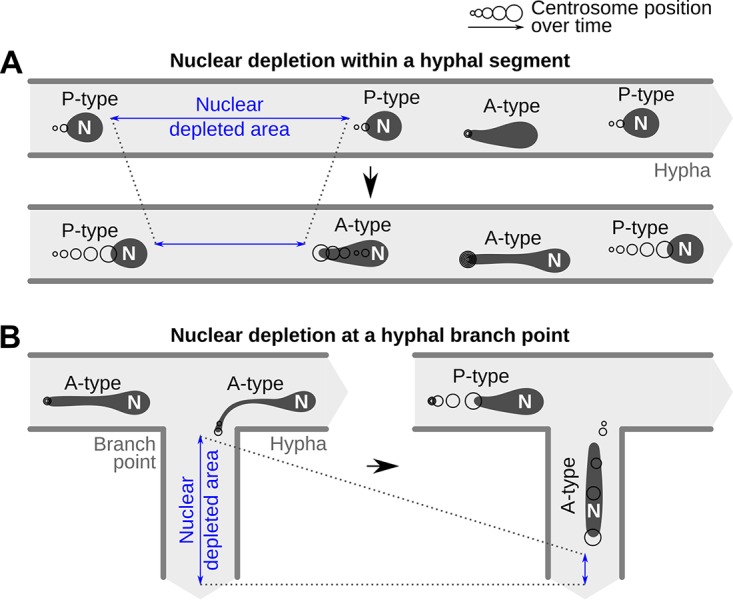
Proposed behavior change from passive (P-type) to active (A-type) movements within *P. palmivora* hyphae. (A) Decrease in nuclear density within a hyphal segment (blue) causes downstream nuclei to anchor until populated by P-type nuclei or to initiate retrograde movement to fill it directly. Anchorage is mediated by the centrosome and results in nuclear stretching. (B) Decrease in nuclear density in a branching hypha (blue) causes nuclei to anchor at the branch point and initiate active movement to enter the branch.

We describe the active, long-distance rerouting of individual nuclei toward nucleus-depleted areas, and our data suggest that such A-type movements contribute to the near-equal spacing of nuclei within germ tubes, infectious hyphae, and axenic mycelium (Fig. 8). We found that A-type movements are impaired by Benomyl, suggesting that they require microtubules. Consistent with our observations, a proper nuclear distribution within A. nidulans and N. crassa hyphae requires microtubules (7, 8), and an additional role of cytoplasmic dynein has been reported (9, 10). Future work will investigate the role of *P. palmivora* dynein in nuclear movement.

Several reports suggest that a local variation in tubulin concentration may affect microtubule polymerization in different model systems. For instance, a local increase in tubulin concentration in neurons may favor nucleation of noncentrosomal microtubule bundles (39). Besides, the onset of mitosis in the fungus A. nidulans triggers a rapid influx of tubulin into the nucleoplasm (40), and local accumulation of tubulin monomers in the nascent spindle region of mitotic nuclei has been reported from Caenorhabditis elegans embryos (41). The tempting speculation that local changes in tubulin concentration favor transition from P- to A-type nuclear trajectories in *P. palmivora* hyphae will require future experimentation.

Equidistant nuclear distribution mechanisms and nuclear migration are likely to occur in other oomycete species, including those not belonging to the family Peronosporaceae. For instance, indirect evidence suggests that nuclear migration occurs in germinating cysts of *Lagenisma coscinodisci*, a diatom parasite from an early-diverging Saprolegniomycetes lineage (42). In this species, a near-globular nucleus migrates from the cyst to the germ tube and concomitantly stretches, reaching a length of up to 10 μm, presumably due to the narrowness of the germ tube (24). Therefore, *P. palmivora* nuclear dynamics may be similar to other oomycetes.

We showed a correlation between active movement of *P. palmivora* nuclei and nuclear stretching. A-type nuclei were stretched up to 30 μm in length upon speed change, irrespective of the diameter of the surrounding hyphae. They revert back to round shape within seconds when returning to P-type trajectory. Nuclear deformability has also been reported in fungi. For instance, during rice infection by Magnaporthe oryzae, migration of the appressorial nucleus within the narrow penetration peg causes nuclear constriction up to 13 μm (43). While extreme constrictions of fungal nuclei were triggered by the narrowness of the surrounding hyphal structures (43), *P. palmivora* nuclei also deformed in hyphae with diameters much larger than their nuclei. Deformability has been associated with a specific nuclear lamina composition and chromatin organization (44). Variation in *lamin* gene expression regulates nuclear stiffness and its deformability in a variety of metazoan cells, including hematopoietic and immune cells (45). *Lamin* overexpression increases nuclear stiffness and reduces deformability (45), while loss of *lamin* gene expression in metazoans resulted in increased nuclear deformability but also in increased fragility (44, 46). Moreover, defects in lamina assembly are responsible for increased cell death and cause a large array of life-threatening laminopathies (44), and *lamin* null mutants show decreased nuclear stiffness. Fungal nuclei are devoid of lamina (47) and lack lamin-encoding genes (31), presumably explaining their deformability. In contrast to fungi, lamin-encoding genes are present in several *Phytophthora* species, including *P. infestans*, P. ramorum, and *P. palmivora* (31, 32). Furthermore, we showed that overexpression of the *P. palmivora laminA* gene alters the nuclear periphery and affects nuclear shape formation in *Phytophthora*.

The centrosome of stretched A-type nuclei always occupies the leading tip, suggesting that nuclear stretching is a result of direct or indirect forces exerted on the centrosome. In agreement with this hypothesis, we observed nuclear tumbling, indicative that A-type movement is polarized. Finally, the astern orientation of centrosomes in P-type nuclei suggests that passive movement may be a transient state that prepares for possible conversions to A type for nuclear rerouting. Taken together, *P. palmivora* is an accessible model organism to study the relationships between nuclear movements, deformability, and rerouting.

Models of lipid bilayer vesicles flowing through circular tubes (48–50) do not predict shapes like those observed in A-type nuclei but primarily reflect red blood cell microcirculation in capillaries and liposome flow through microfluidic compartments. These models therefore do not take into account nucleus-specific parameters such as interactions with the cytoskeleton (51). Besides, simple lipid bilayers may not reflect the biophysical properties of nuclear envelope. For instance, biomechanical models of nuclear shape changes during micropipette aspiration showed that the overall response of an isolated nucleus is highly sensitive to the apparent stiffness of the nuclear lamina (52). We speculate that dramatic stretching minimizes the constraints exerted on nuclei, allowing them to migrate greater distances with limited energy cost. Future work should aim at developing models for hydrodynamic shape adaptations of nuclei to sustain long-distance, flow-independent movements within branched filamentous hyphae.

We documented bidirectional movements of nuclei during cyst germination which were altered by appressorium differentiation. Failure to differentiate an appressorium triggered rerouting of nuclei into additional infection attempts. This suggests that *P. palmivora* nuclear dynamics and developmental transitions are coordinated. Similarly, during rice infection by the fungus *M. oryzae*, one postmitotic nucleus migrates into the developing appressorium, while other nuclei are degraded after entering the conidial cell (3). Later on, the appressorial nucleus migrates down into the primary hypha. Such migration requires a functional transketolase, TKL1, acting as a metabolic checkpoint. Indeed, loss of transketolase function results in depletion of ATP content and slowing down of the TOR signaling pathway (4). Whether an expressed transketolase ortholog of TKL1 in *P. palmivora* (PLTG_04838) (53) is also involved in early infection processes remains to be addressed.

In conclusion, *P. palmivora* is an accessible system which allowed us to highlight commonalities as well as differences in nuclear dynamics between oomycetes and previously studied fungi. Our work sheds new light on nuclear deformability in filamentous microorganisms and uncovers the role of nuclear rerouting to dynamically maintain equal distribution within coenocytic hyphae as well as during plant infection. These findings provide a foundation for hydrodynamic modeling of nuclear shape adaptations in branched hyphal networks. Developing chemical inhibitors targeting the oomycete-specific nuclear movement machinery could stop cyst germination early and therefore represents robust, hard-to-evade targets for durable crop protection against oomycete diseases.

## MATERIALS AND METHODS

### *Phytophthora* strains and growth conditions.

*P. palmivora* Butler isolate LILI (accession no. P16830) was initially isolated from oil palm in Colombia (54) and maintained in the *P. palmivora* collection at the Sainsbury Laboratory (Cambridge, United Kingdom). *P. palmivora* strains were maintained on petri plates of V8 agar (1.5% agar) at 25°C. For maintenance of transformed strains, Geneticin (G418) was added to the medium at a concentration of 100 mg/liter. Mycelium was grown for 5 days in the dark followed by 2 days under constant light conditions. For the latter, plates were left unsealed to remove excess humidity. For production of zoospores, 7-day-old plates were incubated at 4°C for 30 min. Plates were then flooded with 5 ml sterile water and incubated at room temperature for another 30 min. N. benthamiana growth conditions were described previously (53).

### Plasmid construction.

An attR3/attR4 MultiSite Gateway cassette was PCR amplified from pK7m34GW (55) using GoTaq DNA polymerase (Promega UK, Southampton, United Kingdom) with primers NdeI-R3 and NdeI-R4 (see Table S1B in the supplemental material), flanking each side of the cassette with NdeI restriction sites. The MultiSite Gateway cassette was then ligated into pGEM-T Easy vector (Promega UK, Southampton, United Kingdom), and the insert sequence was confirmed by sequencing (Source BioScience, Nottingham, United Kingdom). Thereafter, the cassette was ligated into pTOR vector (56) using the NdeI restriction sites replacing the vector’s Ham34 promoter, multiple cloning site, and Ham34 terminator, resulting in plasmid pTORKm43GW. The orientation of the attR3/attR4 cassette was selected so that promoters inserted using the Gateway cassette will not be in close proximity to the Hsp70 promoter driving the antibiotic selection marker.

pTOR vectors carrying a cassette for Ham34 promoter-driven expression of mTFP1 (cyan) (57), mWasabi (green) (58), mCitrine (yellow) (59), or tdTomato (red) (60) were derived from KpnI-linearized pTORKm43GW. Briefly, Ham34 promoter, Ham34 terminator, and the reporters were amplified individually using primer pairs listed in Table S1B. The final cassettes were assembled by overlap extension PCR and used for In-Fusion cloning (Clontech, Palo Alto, CA, USA) into linearized pTOR-Gateway vectors. An outline of the pTOR-Gateway backbone is shown in Fig. S1.

### Genomic DNA extraction.

Genomic DNA was extracted from axenically grown *P. palmivora* mycelium using a protocol modified from the work of Möller et al. (61). Briefly, samples were incubated in a 1:1 mix of STES buffer (50 mM Tris-HCl, pH 8.0, 10 mM EDTA, 150 mM NaCl, 2% [vol/vol] SDS) and Tris-buffered phenol solution (pH 8.0) for 30 min at 65°C. After phenol-chloroform extraction, nucleic acids were treated with RNase T_1_ (Life Technologies Ltd., Paisley, United Kingdom) for 30 min at 37°C. RNase was removed by phenol-chloroform extraction prior to isopropanol precipitation of genomic DNA.

### Cloning of *Phytophthora* promoters and terminators.

Primers for *P. palmivora* promoters and terminators used in this study were derived from publicly available genomic resources (32) (Table S1B). Constructs were generated using two-step Gateway PCR (Invitrogen, Carlsbad, CA, USA) and subsequently cloned into pDONR221 entry vectors carrying attP4-attP1R or attP2R-attP3 Gateway cassette, respectively. Sequences were confirmed by sequencing (Source BioScience, Nottingham, United Kingdom). The *ubiquitin-conjugating enzyme 2* (*UBC2*) promoter was defined as 1,500 bp upstream of the start codon. Terminator consisted of 500 bp downstream of the *UBC2* stop codon. *LaminA* and *Centrin2* (*CETN2*) promoters were defined as 1,000 bp upstream of the start codon. The nuclear reporter used in this study was obtained by fusing a tandem repeat of Phytophthora sojae *bZIP1* nuclear localization signal (29) to the 5′ end of *mTFP1*. The centrosome reporter was obtained by fusing *mCitrine* to the 5′ end of *P. palmivora CETN2*.

### Generation of transgenic *P. palmivora*.

Transgenic *P. palmivora* was obtained by zoospore electrotransformation using the method from the work of Huitema et al. (62) with the following modifications: for electroporation, 680 μl of high-concentration (>10^6^ zoospores/ml), high-mobility zoospore suspension was mixed with 80 μl of 10× modified Petri’s solution and 40 μl (20 to 80 μg) of plasmid DNA. Electroporation settings were as follows: voltage, 500 V; capacitance, 50 μF; resistance, 800 Ω. After electroporation, zoospore suspensions were diluted to 6 ml with clarified V8 medium and incubated at 25°C for 6 h on a rocking shaker. The encysted zoospore suspension was plated on a 15-cm-diameter plate with selective medium containing 100 mg/liter Geneticin. Transformants were transferred to fresh selective plates up to 10 days after transformation.

### Quantitative reverse transcription-PCR (qRT-PCR) analyses.

Total RNA was extracted from axenically grown *P. palmivora* mycelium containing sporangia (sample MZ) and N. benthamiana roots harvested at 3, 6, 18, 24, 30, and 48 h after inoculation (hai) with *P. palmivora* ARI-td zoospores (63) using the RNeasy plant minikit (Qiagen, Germantown, MD, USA). One microgram was reverse transcribed to generate first-strand cDNA, using the Bio-Rad IScript cDNA synthesis kit according to the manufacturer’s instructions (Bio-Rad, Hercules, CA, USA). RNA quality was assessed by electrophoresis on an agarose gel. Conditions for quantitative PCR were described previously (53).

### Confocal microscopy.

Confocal laser scanning microscopy images were acquired with a Leica SP8 laser-scanning confocal microscope equipped with a Leica HC Fluotar 25× 0.95-numerical-aperture (NA) objective (Leica, Wetzlar, Germany). A white-light laser was used for excitation at 477 nm for mTFP1 (excitation maximum, 462 nm; emission maximum, 492 nm) visualization, 488 nm for mWasabi visualization, 514 nm for mCitrine visualization, and 543 nm for the visualization of tdTomato. Fluorescence acquisition was done sequentially. For time-lapse imaging of infected N. benthamiana roots, seedlings were mounted between a slide and a coverslip in sterile water containing the zoospore suspension. The coverslip was sealed to the slide using a modified Valap sealing (64) composed of a 4:1 mix of paraffin (Sigma-Aldrich, United Kingdom) and lanolin (Sigma-Aldrich, United Kingdom) to prevent dehydration (Fig. S5). Time-lapse imaging of axenically grown mycelium was carried out on plates flooded with 10 ml sterile water. Pictures were analyzed with the ImageJ software (http://imagej.nih.gov/ij/) using the plugin Bio-Formats (https://imagej.net/Bio-Formats). Signal-to-noise ratio was optimized uniformly on time-lapse images series by adjusting minimum and maximum intensity levels. Then, Z-stacks were overlaid using maximum intensity projection and saved in false colors. Videos were generated from overlaid images with ffmeg (https://ffmpeg.org/).

### Measurements of nuclear speed and shape.

Nuclear position was defined as the coordinates of its centroid, obtained from the ImageJ software. Instantaneous speed was plotted using the R software (https://www.r-project.org/) and ggplot2 package (https://ggplot2.tidyverse.org/). Nuclear shape was defined as the ratio (*R_F_*) between Feret’s maximum diameter (maximum caliper) and Feret’s minimum diameter (minimum caliper). Nuclei were considered round when *R_F_* was <2, while larger *R_F_* values indicated stretched nuclei.
