# Supplementary material for: Hydrodynamic Shape Changes Underpin Nuclear Rerouting in Branched Hyphae of an Oomycete Pathogen
Source: mBio. 2019 Oct 1;10(5):e01516-19. doi: 10.1128/mBio.01516-19 (PMC6775453; doi:10.1128/mBio.01516-19)
Supplement: FIG S1 [file mBio.01516-19-sf001.pdf]

# Figure S1

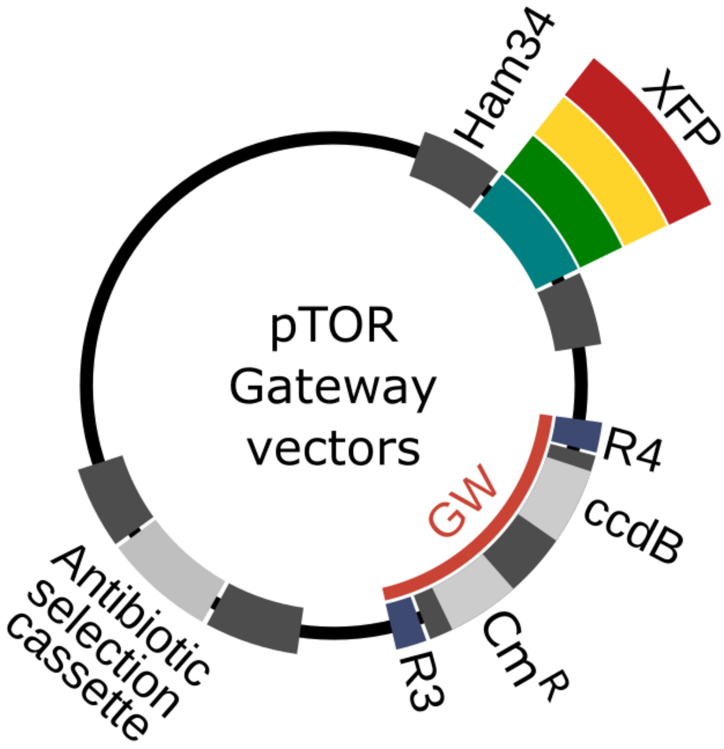


**Figure S1. Schematic representation of the pTOR-Gateway vector series.** A multisite attR4/attR3 Gateway insertion cassette allows for rapid testing of multiple promoter-reporter constructs. Adjacent is a cassette for constitutive expression of a cytoplasmic fluorescent reporter (either mTFP1, mWasabi, mCitrine or tdTomato) under the control of *Bremia lactucae* promoter Ham34. Vectors carry neomycin phosphotransferase (*nptII*) selectable marker.
