# Supplementary material for: Hydrodynamic Shape Changes Underpin Nuclear Rerouting in Branched Hyphae of an Oomycete Pathogen
Source: mBio. 2019 Oct 1;10(5):e01516-19. doi: 10.1128/mBio.01516-19 (PMC6775453; doi:10.1128/mBio.01516-19)
Supplement: FIG S2 [file mBio.01516-19-sf002.pdf]

Figure S2

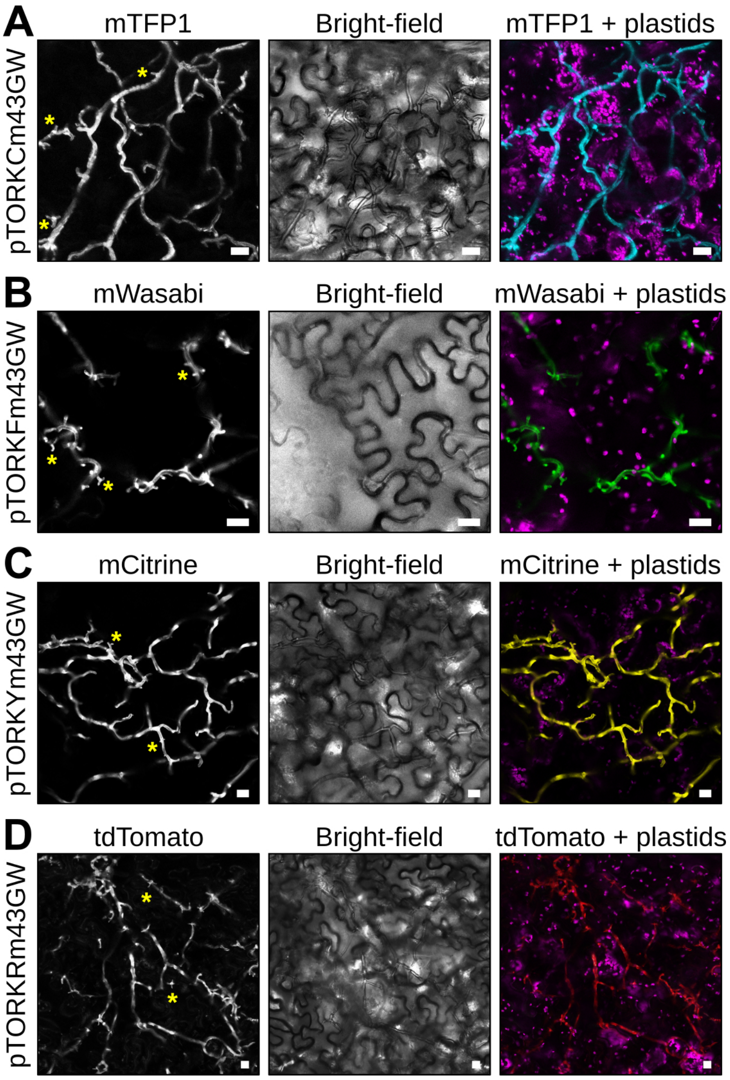

Figure S2. Growth habit of transgenic *P. palmivora* strains carrying empty pTOR-Gateway vectors on *N. benthamiana* leaves. (A-D) Leaves from 4-week-old *N. benthamiana* plants were inoculated with mycelium plugs of transgenic *P. palmivora* strains carrying empty pTOR-Gateway vectors. Fluorescence was monitored within leaf tissues after two days. Representative images of areas infected with mycelium expressing mTFP1 **(A)**, mWasabi **(B)**, mCitrine **(C)** and tdTomato **(D)** are shown. Yellow asterisks indicate haustoria. Scale bar is 10 µm.
