# Supplementary material for: Hydrodynamic Shape Changes Underpin Nuclear Rerouting in Branched Hyphae of an Oomycete Pathogen
Source: mBio. 2019 Oct 1;10(5):e01516-19. doi: 10.1128/mBio.01516-19 (PMC6775453; doi:10.1128/mBio.01516-19)
Supplement: FIG S3 [file mBio.01516-19-sf003.pdf]

Figure S3

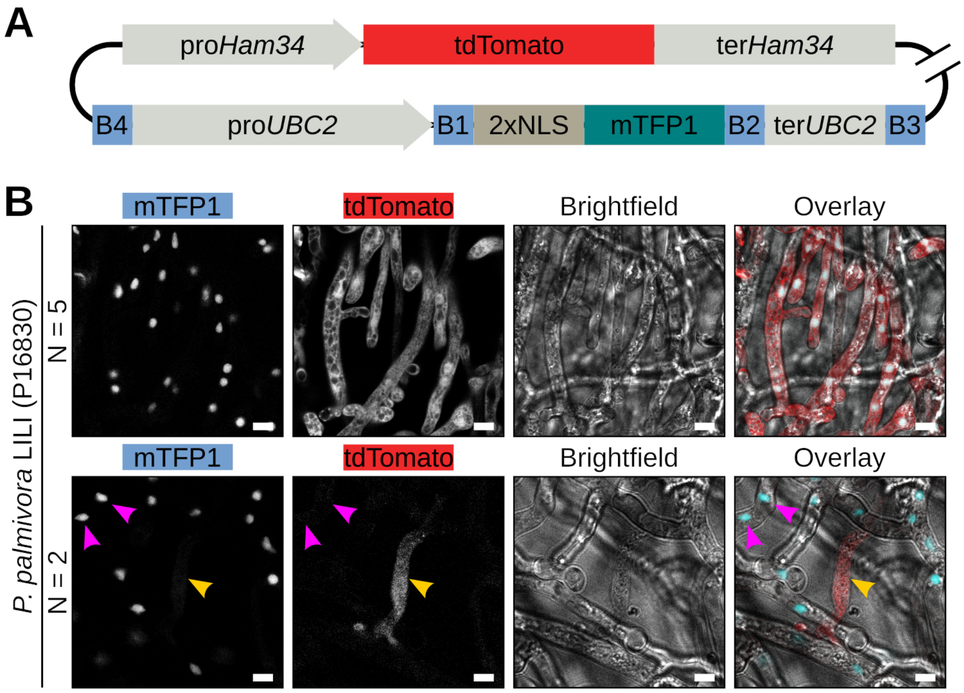

Figure S3. Dual labelling of *P. palmivora* hyphae and nuclei. (A) Schematic view of the construct used for dual labelling of nuclei and hyphae in *P. palmivora* strain LILI-td-NT. Backbone elements are not represented. **(B)** Representative pictures of a transformant expressing tdTomato as well as a nuclear-localized mTFP1 (NLS:mTFP1) driven by the *P. palmivora ubiquitin-conjugating enzyme 2* (*UBC2*) native promoter. Representative pictures of transformants expressing both markers as well as rare cases of transformants expressing either tdTomato or mTFP1 in distinct hyphae. Scale bar is 10 µm.
