# Supplementary material for: Hydrodynamic Shape Changes Underpin Nuclear Rerouting in Branched Hyphae of an Oomycete Pathogen
Source: mBio. 2019 Oct 1;10(5):e01516-19. doi: 10.1128/mBio.01516-19 (PMC6775453; doi:10.1128/mBio.01516-19)
Supplement: FIG S4 [file mBio.01516-19-sf004.pdf]

Figure S4

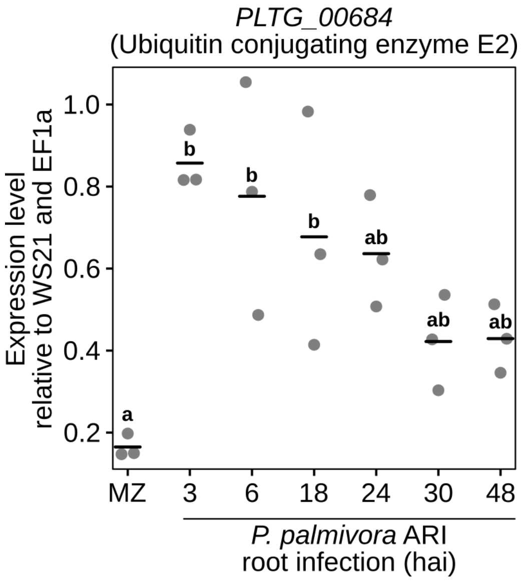

Figure S4. *UBC2* transcript levels during *N. benthamiana* root infection. *N. benthamiana* roots were inoculated with zoospores from the transgenic *P. palmivora* strain ARI-tdTomato (Le Fevre *et al*, 2016) and harvested at different times corresponding to early infection (3-6 hours), biotrophy (18-24 hai) and necrotrophy (30-48 hai). Expression data are given relative to *P. palmivora WS21* and *EF1α* reference genes. Statistical significance was assessed using one-way ANOVA and Tukey's HSD test ($P < 0.05$). MZ: axenically grown mycelium with sporangia.
