# Supplementary material for: Hydrodynamic Shape Changes Underpin Nuclear Rerouting in Branched Hyphae of an Oomycete Pathogen
Source: mBio. 2019 Oct 1;10(5):e01516-19. doi: 10.1128/mBio.01516-19 (PMC6775453; doi:10.1128/mBio.01516-19)
Supplement: TABLE S1 [file mBio.01516-19-st001.pdf]

Table S1

Plasmid name	Fluorophore	Selection
pTORKm43GW	*None*	G418
pTORKCm43GW	mTFP1	G418
pTORKFm43GW	mWasabi	G418
pTORKYm43GW	mCitrine	G418
pTORKRm43GW	tdTomato	G418

Table S1A. pTOR-Gateway vectors. pTOR-Gateway vectors follow naming conventions used for Gateway vectors. K indicates *nptII*, while C, F, Y and R stand for cyan, green, yellow and red fluorescence, respectively. The multi-site Gateway cassette carries attR4 and attR3 sites and hence was arbitrarily named m43GW in the absence of a T-DNA left border to define cassette orientation.

Primer name	Sequence (5′ -> 3′)
**Quantitative RT-PCR**	
Lili-UBC2-F2	AACATTTACTTCCCGGCCGAC
Lili-UBC2-R2	CAGGGCTCCATTGGTCCTTCA
Lili-EF1a-F1	GCAACATGCCGTGGTACAAG
Lili-EF1a-R1	CTTAGGGGCGTTCAGGTTGT
Lili-WS21-F1	CTCCAGAACGTGTACATTCG
Lili-WS21-R1	TGGCACCCTTCTCCTCGG
**pTOR-Gateway backbone**	
NdeI-R3	ACGCATATGTGAATTATCAACTATGTATAAT
NdeI-R4	ACGCATATGCCAAGCTATCAACTTTGTATAG
Ham34pro_IFF	AGGGAACAAAAGCTGGTACCTCTGATGGACAAAGGGTC
Ham34pro_XFP_R	CTCCTCGCCCTTGCTCACCATGGTATCGATACCGTCG
XFP_F	GTGAGCAAGGGCGAGGAG
Ham34ter_XFP_R	CAGCTCGACCTTCGGCCTACTTGTACAGCTCGTCCATG
Ham34pro_R	GGTATCGATACCGTCGACCT
Ham34ter_F	GCCGAAGGTCGAGCTGTGTG
Ham34ter_IFR	GGTATAATGGGCCCGGTACCTGGTTGGTTTACGATAAATAAAG
**Nuclear reporter driven by *UBC2* promoter**	
UBC2pro_2B4	TATAGAAAAGTTGGCCCTTACTGGTCTCCGACACGAGC
UBC2pro_2B1R	TTTGTACAAACTTGCTTGCTCTGTTCCTCCTCTCCTTCG
UBC2ter_2B2R	TTGTACAAAGTGGAGCACGCTAGGAGTAGGAGATCTTC
UBC2ter_2B3	TAATAAAGTTGATCATAAAACAATGTGACAATAGCG
WS21ter_2B2R	TTGTACAAAGTGGGCGCGTTCATTAGTTGACAGCTTGG
WS21ter_2B3	TAATAAAGTTGTTGGCTGAGGCGCAGTTCTGTAGAA
NLS_2B1	AAAAAAGCAGGCTATGCACAAGCGCAAGCGCG
NLS_mTFP1_R	CTCCTCGCCCTTGCTCACGCGACGACGGACCTTGGTGTCG
mTFP1_2B2	CAAGAAAGCTGGGTCTACTTGTACAGCTCGTCCATG
**CETN2 reporter**	
Cit_CETN2_R	TCGCGAGCCATAAGCGGAAGACATCTTGTACAGCTCGTCCAT
CETN2_F	ATGTCTTCCGCTTATGGCTCGCGA
CETN2_Ham34t_R	GTGCACACAGCTCGACCTTCGGCTTAGTACAGACTAGTCTTC
CETN2pro_F	CACGGACAATGCTTGGATCT
CETN2pro-SCA_R	gcctcgcccttgctcaccatCACTGCGTCTCTTGT
SCA_CE_R	GAGCCATAAGCGGAAGACATCTTGTACAGCTCGTC
CE_CEter_R	GTATTGTCTCCGTTTGCCCCTTAGTACAGACTAG
**LaminA reporter**	
mCitrine_LamA_R	CTTTGTGTTGGGCGTCGTGACAGCCACCTTGTACAGCTCGTCCAT
LamA_F	GTGGCTGTCACGACGCCCAACACAA
LamA_Ham34ter_R	ACAGCTCGACCTTCGGCTTACATCATGCCGCAGTTTCCAGAGGGAGGAGCCT
LamA_Ham34ter_F	GCATGATGTAAGCCGAAGGTCGAGCTGT
LamApro_IFF	AGGGAACAAAAGCTGGTACCAGGCAAAGGAGAAGCGTG
LamApro_mCitrine_R	GCTCCTCGCCCTTGCTCACCATGATGGACTCGAACACGTGCG

Table S1B. Primers used in this study.
