# Supplementary material for: Hydrodynamic Shape Changes Underpin Nuclear Rerouting in Branched Hyphae of an Oomycete Pathogen
Source: mBio. 2019 Oct 1;10(5):e01516-19. doi: 10.1128/mBio.01516-19 (PMC6775453; doi:10.1128/mBio.01516-19)
Supplement: FIG S5 [file mBio.01516-19-sf005.pdf]

Figure S5

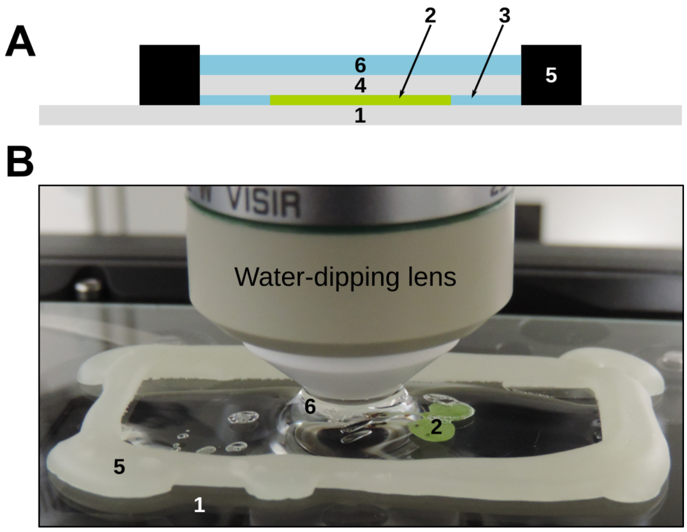

Figure S5. Experimental set-up used for time-lapse imaging of infected *N. benthamiana* roots. (A) Schematic representation of the experimental set-up. A one-week-old *N. benthamiana* seedling (2) is mounted in a liquid compartment (3) between a slide (1) and a coverslip (4). The edges of the coverslip are sealed with a 4:1 mix of paraffin and lanolin (5). The upper part of the coverslip is filled with water for use with a water-dipping objective. **(B)** Representative image of the experimental set-up. Numbers refer to the same elements as described previously.
