# Supplementary material for: Hydrodynamic Shape Changes Underpin Nuclear Rerouting in Branched Hyphae of an Oomycete Pathogen
Source: mBio. 2019 Oct 1;10(5):e01516-19. doi: 10.1128/mBio.01516-19 (PMC6775453; doi:10.1128/mBio.01516-19)
Supplement: FIG S6 [file mBio.01516-19-sf006.pdf]

Figure S6

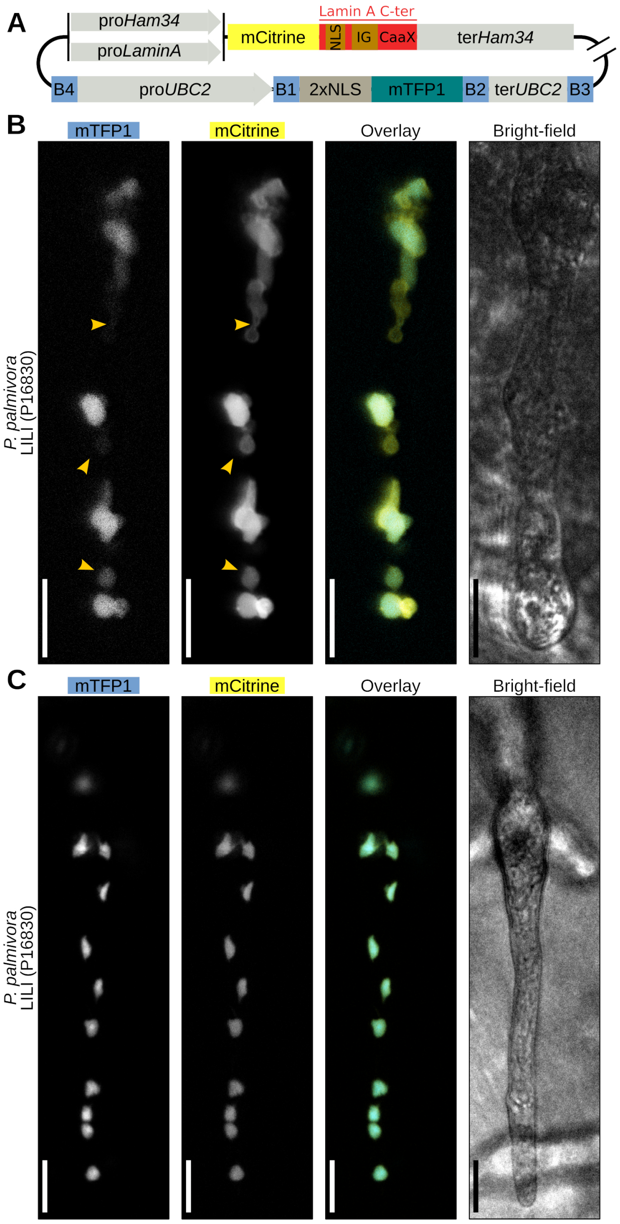

Figure S6. Generation of a *P. palmivora* lamin A reporter. (A-C) Transformation of *P. palmivora* LILI with a construct for constitutive **(A-B)** or native **(C-D)** expression of a mCitrine:LamA-Cter reporter. **(A)** Schematic view of the construct used for constitutive or native expression of the lamin reporter together with a nuclear-localized mTFP1. Backbone elements are not represented. **(B)** Representative pictures of the bubbling phenotype observed upon constitutive expression of the lamin reporter. Arrowheads indicate bubbling nuclei. Scale bar is 10 µm. **(C)** Representative pictures of a hyphal segment upon native expression of the lamin reporter, showing absence of nuclear bubbling. Arrowheads indicate bubbling nuclei. Scale bar is 10 µm.
