# Supplementary material for: Hydrodynamic Shape Changes Underpin Nuclear Rerouting in Branched Hyphae of an Oomycete Pathogen
Source: mBio. 2019 Oct 1;10(5):e01516-19. doi: 10.1128/mBio.01516-19 (PMC6775453; doi:10.1128/mBio.01516-19)
Supplement: FIG S7 [file mBio.01516-19-sf007.pdf]

Figure S7

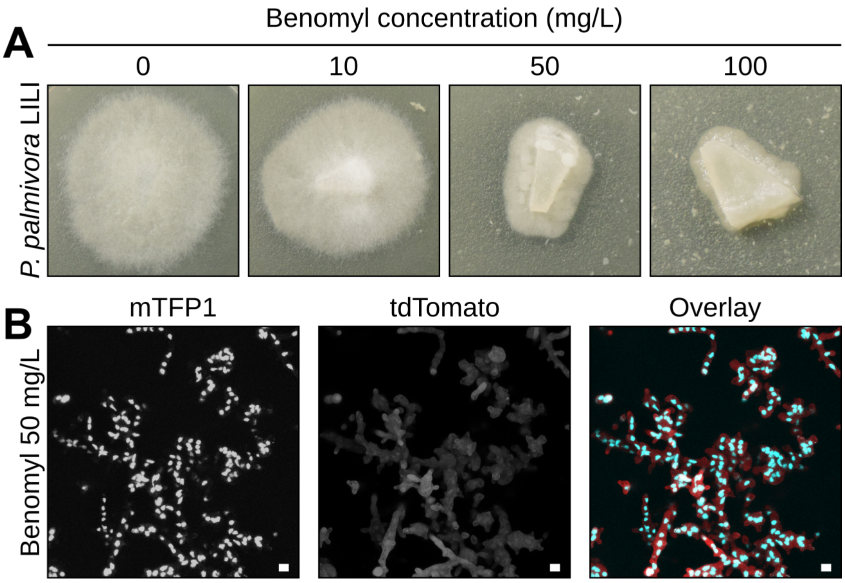

Figure S7. Effect of antimicrotubule drug Benomyl on *P. palmivora* growth. (A) Representative pictures of *P. palmivora* LILI-td-NT mycelium growing on V8 agar plates supplemented or not with 10, 50 or 100 mg/L Benomyl. **(B)** Confocal imaging of *P. palmivora* LILI-td-NT hyphae grown on V8 agar plates supplemented with 50 mg/L Benomyl. Scale bar is 10 µm.
