# Supplementary material for: Hydrodynamic Shape Changes Underpin Nuclear Rerouting in Branched Hyphae of an Oomycete Pathogen
Source: mBio. 2019 Oct 1;10(5):e01516-19. doi: 10.1128/mBio.01516-19 (PMC6775453; doi:10.1128/mBio.01516-19)
Supplement: FIG S8 [file mBio.01516-19-sf008.pdf]

Figure S8

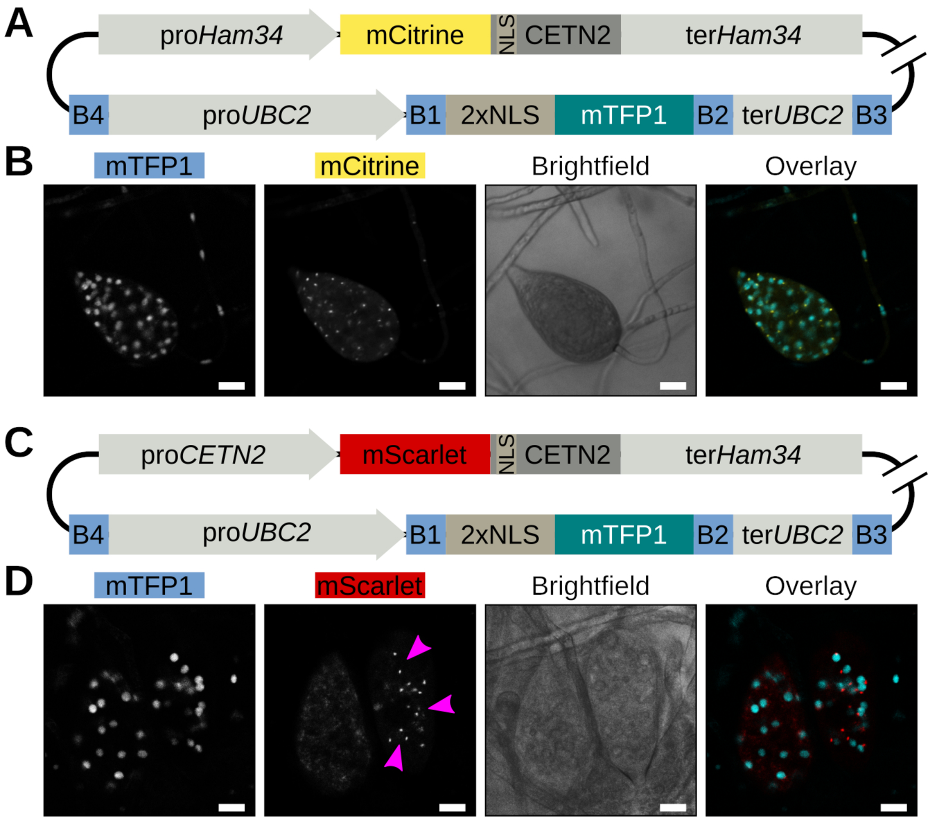

Figure S8. Generation of a *P. palmivora* Centrin 2 (CETN2) reporter. (A) Schematic view of the construct for Ham34-promoter-driven expression of a mCitrine:CETN2 reporter together with a nuclear-localized mTFP1. Backbone elements are not represented. **(B)** Confocal imaging of a sporangium from a *P. palmivora* LILI-NT-Ce transgenics expressing the construct shown in **(A)**. Scale bar is 10 µm. **(C)** Schematic view of the construct for CETN2-promoter-driven expression of a mScarlet:CETN2 reporter together with a constitutively expressed nuclear-localized mTFP1. **(D)** Confocal imaging of a sporangium from a transgenic *P. palmivora* strain expressing the construct shown in **(C)**. Scale bar is 10 µm.
