# Supplementary material for: Hydrodynamic Shape Changes Underpin Nuclear Rerouting in Branched Hyphae of an Oomycete Pathogen
Source: mBio. 2019 Oct 1;10(5):e01516-19. doi: 10.1128/mBio.01516-19 (PMC6775453; doi:10.1128/mBio.01516-19)
Supplement: FIG S9 [file mBio.01516-19-sf009.pdf]

Figure S9

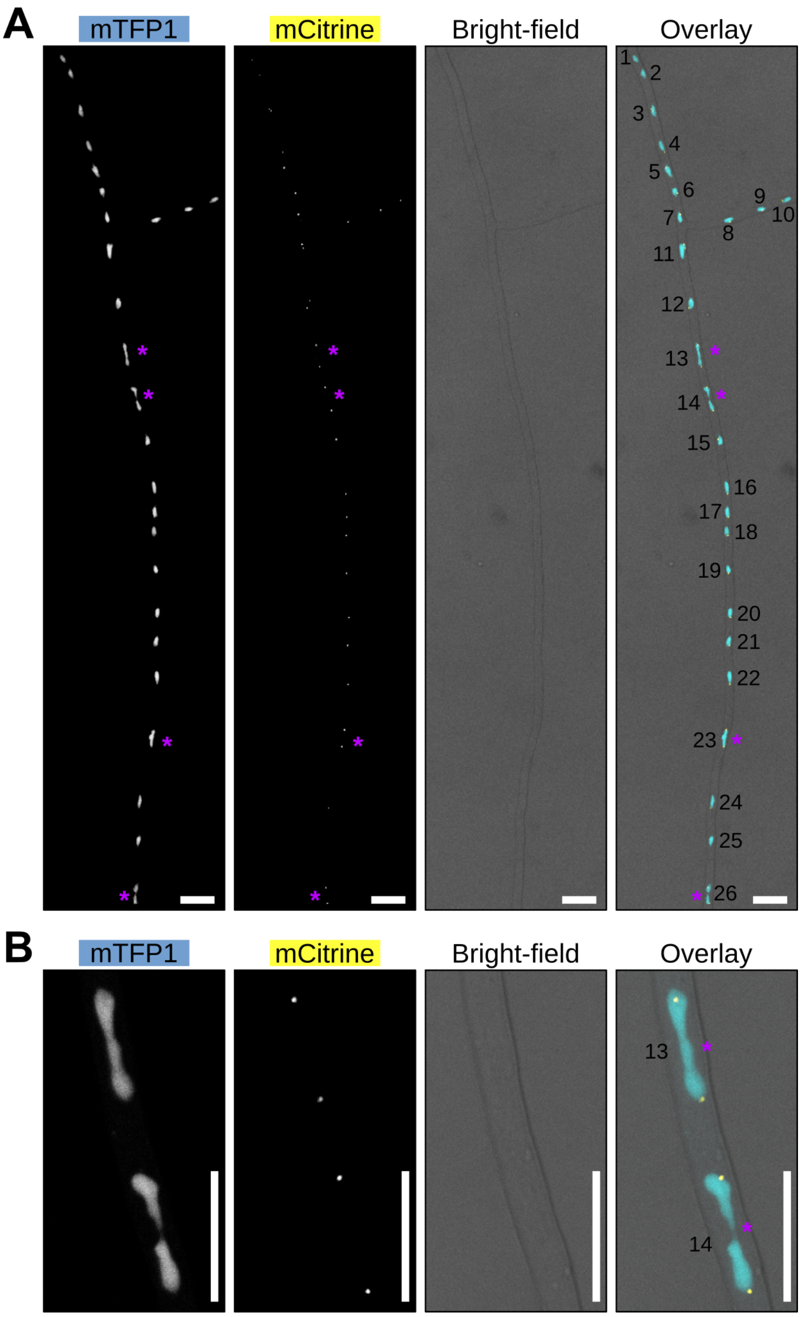

Figure S9. Frequency of centrosome duplication within *P. palmivora* hyphae. (A-B) Representative pictures of axenically-grown hyphae from the transgenic *P. palmivora* LILI-NT-Ce strain growing on V8 medium. **(A)** Distribution of nuclei and Centrin 2 (CETN2)-labelled centrosomes within an hyphal segment. Asterisks indicate nuclei with duplicated centrosomes. **(B)** Magnified views of nuclei 13 and 14. Scale bar is 10 µm.
